# Association between toe flexor strength and spatiotemporal gait parameters in community-dwelling older people

**DOI:** 10.1186/1743-0003-11-143

**Published:** 2014-10-08

**Authors:** Shogo Misu, Takehiko Doi, Tsuyoshi Asai, Ryuichi Sawa, Kota Tsutsumimoto, Sho Nakakubo, Minoru Yamada, Rei Ono

**Affiliations:** Kobe City Hospital Organization, Kobe City Medical Center, West Hospital, 2-4 Ichibancho,Nagata-ku,, Kobe, Hyogo 653-0013 Japan; Department of Community Health Sciences, KKobe University Graduate School of Health Sciences, 7-10-2 Tomogaoka,Suma-ku, Kobe, Hyogo 654-0142 Japan; Section for Health Promotion, Department for Research and Development to Support Independent Life of Elderly, Center for Gerontology and Social Science, National Center for Geriatrics and Gerontology, 35 Gengo, Morioka, Obu, Aichi 474-8511; Japan Society for the Promotion of Science, Kojimachi Business Center Building, 5-3-1 Kojimachi, Chiyoda-ku, Tokyo 102-0083 Japan; Research Institute, National Center for Geriatrics and Gerontology, 35 Gengo, Morioka, Obu, Aichi 474-8511; Department of Physical Therapy, Faculty of Rehabilitation, Kobe gakuin University, 518 Arise, Ikawadani-cho, Nishi-ku, Kobe, Hyogo 651-2180 Japan; Department of Rehabilitation Sciences, Kobe University Graduate School of Health Sciences, 7-10-2 Tomogaoka,Suma-ku,, Kobe, Hyogo 651-2180 Japan; Graduate School of Comprehensive Human Sciences, University of Tsukuba,, 3-29-1 Otsuka, Bunkyo-ku, Tokyo, 112-0012 Japan

**Keywords:** Gait, Toe flexor strength, Spatiotemporal gait parameter, Community-dwelling older people

## Abstract

**Background:**

The toe flexor muscles perform a crucial function to control foot movement and assist with propulsive force when walking. However, the association between toe flexor strength and spatio-temporal gait parameters is largely unknown. Spatiotemporal gait parameters represent gait characteristics, and are good measures of the functional status and degree of safe ambulation among community-dwelling older adults. Herein, we examined the association between the toe flexor strength and spatiotemporal gait parameters in community-dwelling older adults.

**Methods:**

Ninety-three community-dwelling older people (mean age: 73.2 ± 4.2 years, 53 women) participated in this study. The strength of the toe flexor muscles was assessed using a toe strength measuring instrument and a strain gauge. The measurements were performed once on each foot, and the average of the right and left was used in the analysis. Gait analysis was performed on a 15-m walkway under usual- and fast-pace conditions. The medial 10-m walking time was measured and walking speed was calculated. Acceleration and angular velocity of the right heel were measured using a wireless miniature sensor unit and used to compute cadence, percent of swing time in gait cycle (%swing time), and stride length.

**Results:**

In multiple regression analyses adjusted for age, sex, body height, body weight, and hand grip strength, no associations between toe flexor strength and spatiotemporal gait parameters at usual pace were found. Conversely, under the fast-pace condition, decreased toe flexor strength was significantly associated with slower walking speed (*β* = 0.22, *p* = 0.049), lower%swing time (*β* = 0.34, *p* = 0.009), and shorter stride length (*β* = 0.22, *p* = 0.011) after adjustment.

**Conclusion:**

In community-dwelling older people, decreased strength of toe flexor was correlated with slower walking speed, shorter periods of single-limb support phase, and shorter stride length during fast-pace walking. These data provide further support for an important role of toe flexor muscles in walking.

**Electronic supplementary material:**

The online version of this article (doi:10.1186/1743-0003-11-143) contains supplementary material, which is available to authorized users.

## Background

During walking, the foot is the only source of direct contact with the ground. The foot provides mechanical support for the body and sensory information regarding body position, which contributes to maintaining stability and providing forward propulsion. The toe flexor muscles perform a crucial function to control foot movement and assist with the propulsive force when walking. This is achieved by the contraction of the muscles at the late stance phase in the gait cycle [1, 2]. By determining conditions for equilibrium from ground force distribution and anthropometrical foot data, it was estimated that the muscles of the flexor hallucis longus, brevis, flexor digitorum longus and brevis exert forces of approximately 52%, 36%, 9% and 13% of body weight during propulsion, respectively [3].

The association between toe flexor strength and objective gait parameters remains unclear, although the role of the toe flexor for gait is considered important. Various methods with high reliability have been developed for evaluating the strength of toe flexor muscles, including the paper grip test for measuring the forces under the toes while subjects push down a force-plate using their toes, hand-held dynamometry, and the method using a foot-gripping force meter including a strain-gauge [4–6]. Using these methods, it was demonstrated that aging was associated with reduced toe flexion strength [4, 5], and that toe deformities were associated with weak toe flexor strength [7, 8]. In addition, older people with reduced toe flexor strength had impaired balance function and a higher risk of fall [7, 9–11]. Nevertheless, there are few studies that investigated the association between toe flexor strength and gait parameters. Menz et al. [12] reported that toe flexor strength was associated with plantar forces and pressures during walking in older people who resided in a retirement village. Spink et al. [10] reported that hallux plantar flexion strength was associated with walking speed at a normal pace in community-dwelling older people who had an elevated risk of falling and self-reported disabling foot pain. However, these studies did not include spatiotemporal gait parameters except for walking speed. Thus, the effects of toe flexor strength to spatiotemporal gait parameters (e.g., cadence, swing time, and stride length) remain unclear. Spatiotemporal gait parameters are good measures of the functional status and degree of safe ambulation among community-dwelling older adults [1]. Given the function of the toe flexor muscle during gait, we hypothesized that toe flexor strength would be associated with spatiotemporal gait parameters.

The aim of this study was to determine the association between the toe flexor strength and spatiotemporal gait parameters during usual-pace walking and fast-pace walking in community-dwelling older adults. Walking at a fast speed (fast-pace walking) may place a greater demand on physiological systems, and thus provide a good indication of their function [13–15].

## Methods

### Participants

One hundred and twenty community-dwelling older people, aged over 60 years, were recruited through a community organization for older people. Eligibility criteria for this study were that subjects could walk over 15 m independently without the use of a gait aid and that their feet did not have any orthopedic anomalies that would make it difficult to perform the measurement of toe flexor strength. Participants were excluded if they had neurological disease that would affect gait (e.g., stroke or Parkinson’s disease), or cognitive impairment (Japanese version of Rapid Dementia Screening Test score < 8) [16, 17]. Ninety-three people met the criteria and participated in this study. The Research Ethics Committee of the Kobe University Graduate School of Health Science approved the study, and all subjects participating in the study provided informed consent according to the ethical standards set forth in the declaration of Helsinki.

### Toe flexor strength and other measures

**Figure 1:**
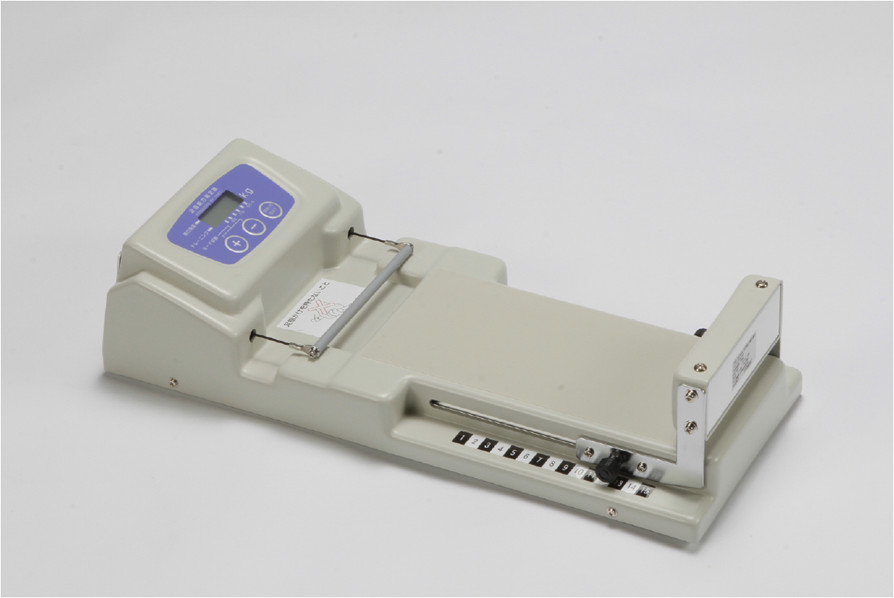
**Toe strength measuring instrument.**

The strength of the toe flexor muscles was assessed using a toe strength measuring instrument (T.K.K. 3361; Takei Scientific Instruments, Niigata, Japan) (Figure 1). The apparatus uses a strain gauge to measure the gripping force of the toe flexor muscles. The resolution performance is 0.1 kg. Subjects sat on a chair with the test foot placed on the instrument and the distal phalanx of the great toe and the second to fifth middle phalanxes attached to the gripping bar. Their hip, knee, and ankle were kept at 90 degrees. After sufficient familiarization, subjects were instructed to grip the bar as hard as possible without any other movement of their lower extremity. The measurements were completed once on each foot, and the average of the right and left was used in the analysis. We also measured hand grip strength as the representative measure of general muscle strength to explore the independent effect of toe flexor strength to gait parameters. Hand grip strength was determined using a hand grip dynamometer (T.K.K. 5401; Takei Scientific Instruments). The measurements were completed once on each hand, and the average was used in the analysis.

We assessed the following background characteristics using a questionnaire: age, sex, medical conditions, number of medications, history of falls and participant’s life space. The participants’ medical conditions, number of medications and fall events during the past 12 months was determined at interviews. Life space was assessed using a life-space assessment (LSA) questionnaire. An LSA assesses frequency of movement (how many days within a week) during the 4 weeks prior to assessment for five different life-space levels [18]. From a total of 20 items, a composite score in the range of 0–120 is computed. Higher scores indicate unrestricted life-space mobility. The reliability and validity of an LSA in community-dwelling older adults have been previously reported [18]. An LSA reflects physical activity status indirectly in elderly people [19]. The anthropometric index (body weight and body height) was also obtained from physical examination.

### Gait procedure and apparatus

Participants were instructed to walk along a 15-m smooth, horizontal walkway including a 2.5-m space before each end of the walkway for acceleration and deceleration while wearing appropriate-sized shoes checked before each gait assessment. Two walking conditions were measured successively in nonrandomized order to avoid the lying effect whereby the usual-pace would be contaminated by the fast-pace condition: subjects first walked at a self-selected usual pace and then walked as fast as possible without running [13, 20–22]. The measurements were performed over the medial 10-m distance. The 10-m walking time was measured, and walking speed was expressed in meters per second.

The wireless miniature sensor unit containing a three-axis accelerometer and a three-axis gyroscope (MVP-RF8; MicroStone, Nagano, Japan) was attached to the posterior surface of the right heel using surgical tape to compute spatiotemporal gait parameters by identifying heel contact and toe off. The miniature sensor unit minimizes restrictions of walking movements. All signals were sampled at 200 Hz and synchronously wirelessly transferred to a personal computer via a Bluetooth personal area network.

### Data analysis

Signal processing was performed with commercially available software (MATLAB, Release 2011b; MathWorks, Natick, MA, USA). Before the analysis, all acceleration and angular velocity data were low-pass filtered with a cutoff frequency of 20 Hz. The temporal gait parameters (cadence and percent of swing time in gait cycle; %swing time) were then determined. On the basis of pilot testing, a heel-contact event was identified as a vertical acceleration peak, while a toe-off event was identified as the maximum heel angular velocity in the sagittal plane. These events were used to calculate temporal gait parameters. Percent of swing time were averaged from five consecutive gait cycles, and were used in the analysis. The average stride length was determined by multiplying gait speed by mean stride time.

### Statistical analysis

Variability of toe flexor strength was transformed into a normal distribution using log transformations because of a right skewed distribution. To examine the association between toe flexor strength and spatiotemporal gait parameters at both usual and fast pace, Pearson correlation coefficients were first computed. Forced-entry multiple regression analyses were then used to clarify the associations between toe flexor strength and spatiotemporal gait parameters independently of confounders. Within participants characteristics measures, variables associated with the strength of toe flexor (*p* < 0.10) in univariate analysis were forced to include as confounders. The overall statistical significance level was set at 0.05. All statistical analyses were performed using JMP9.0 J software (SAS Institute Japan, Tokyo, Japan).

## Results

The characteristics of the subjects and spatiotemporal gait parameters are summarized in Table 1. The mean ± standard deviation (S.D.) of age in our sample was 73.2 ± 4.2 years, and 57.4% of the sample was female. The mean ± S.D. of walking speed at usual and fast pace were 1.42 ± 0.19 m/s and 1.72 ± 0.23 m/s, respectively.

**Table 1:** **Subject characteristics and spatiotemporal gait parameters**

		Mean ± S.D.	Range
Age, years		73.2 ± 4.2	63–88
Sex, women, n (%)		53 (57.4)	
Body weight, kg		56.0 ± 10.8	37.1–88.4
Body height, cm		155.3 ± 8.8	139.7–175.4
BMI, kg/m^2^		23.1 ± 3.2	15.1–35.9
LSA		78.8 ± 18.1	44–120
Falling in past 12 months, n (%)		19 (20.4)	
Medical conditions, n (%)			
	Osteoarthritis	15 (16.1)	
	Rheumatoid arthritis	1 (1.1)	
	Hypertension	41 (44.1)	
	Diabetes mellitus	7 (7.5)	
	Heart disease	8 (8.7)	
Number of medications, n		2.0 ± 1.6	0–6
Strength of hand grip, kg		27.7 ± 6.7	15.3–42.5
Strength of toe flexor, kg		7.5 ± 4.5	1.4–26.0
Usual-pace walking			
	Walking speed, m/s	1.42 ± 0.19	0.91–1.95
	Cadence, steps/min	125.9 ± 10.2	99.2–145.8
	%Swing time	40.77 ± 1.94	34.57–45.57
	Stride length, m	1.35 ± 0.14	1.02–1.67
Fast-pace waking			
	Walking speed, m/s	1.72 ± 0.23	1.04 – 2.35
	Cadence, steps/min	137.8 ± 11.9	111.1–168.1
	%Swing time	41.61 ± 2.07	35.61–46.50
	Stride length, m	1.50 ± 0.17	1.02–1.84

Values are means ± standard deviation or number of subjects (percentages) as indicated. Spatiotemporal gait parameters were averaged from five consecutive gait cycles. *S.D.* = Standard deviation; *BMI* = Body mass index; *LSA* = Life-space assessment; %*Swing time* = Percent of swing time in gait cycle.

**Figure 2:**
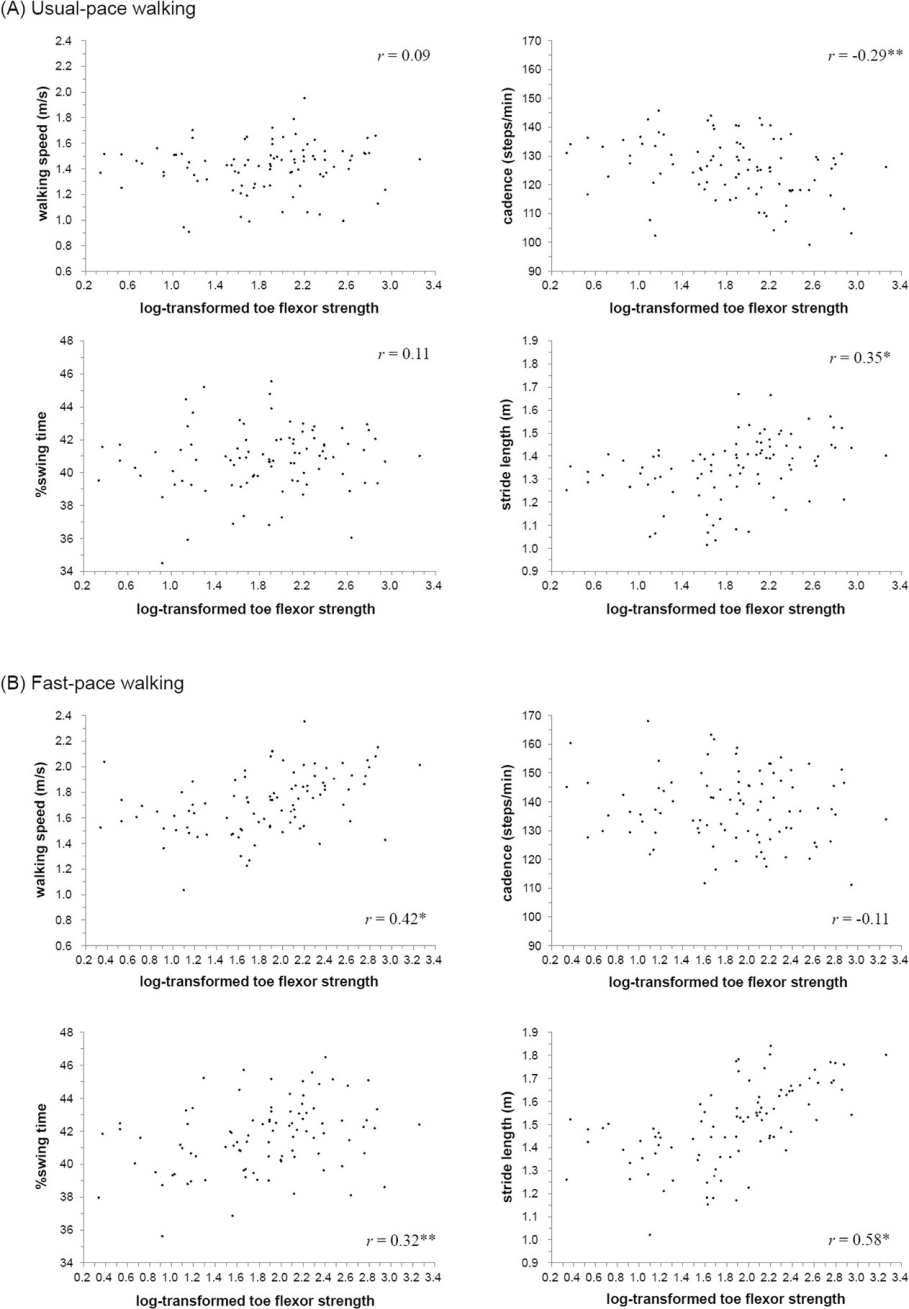
**Bivariate correlation between strength of toe flexor and spatiotemporal gait parameters during usual-pace walking (A) and during fast-pace walking (B).** Variability of toe flexor strength was performed by log transformations. The described *r* value represents Pearson correlation coefficients (* *p* < 0.001, ** *p* < 0.01, *** *p* < 0.05).

Correlation coefficients representing the association between toe flexor strength and spatiotemporal gait parameters are displayed in Figure 2. For the usual-pace condition, the strength of the toe flexor was negatively associated with cadence (*r* = -0.29) and positively associated with stride length (*r* = 0.31) (Figure 2A). For the fast-pace condition, the strength of the toe flexor was positively associated with walking speed (*r* = 0.42), %swing time (*r* = 0.32), and stride length (*r* = 0.58) (Figure 2B).

Table 2 shows the multivariable adjusted associations of spatiotemporal parameters with toe flexor strength. Age, sex, body weight, body height, and hand grip strength were entered into the regression models as confounders as those variables were associated with the strength of toe flexor (*p* < 0.10) in the univariate analysis. After adjustment, no gait parameters at a usual pace were associated with toe flexor strength. Conversely, walking speed (*β* = 0.22, *p* = 0.049),%swing time (*β* = 0.34, *p* = 0.009), and stride length (*β* = 0.22, *p* = 0.011) at a fast pace were significantly associated with toe flexor strength.

**Table 2:** **Multivariable regression – cross-sectional effect of toe flexor strength on spatiotemporal gait parameters at usual- and fast-pace walking**

		Adjusted *R*^*2*^	*β*	*p* value
Usual-pace walking				
	Walking speed (m/s)	0.07	0.10	0.456
	Cadence (steps/min)	0.27	-0.01	0.934
	%Swing time	0.04	0.25	0.057
	Stride length (m)	0.23	0.14	0.233
Fast-pace walking				
	Walking speed (m/s)	0.29	0.22	0.049*
	Cadence (steps/min)	0.10	-0.13	0.623
	%Swing time	0.09	0.34	0.009*
	Stride length (m)	0.57	0.22	0.011*

All models adjusted for age, sex, weight, height, and strength of hand grip. Adjusted *R*^2^ = proportion of variance in each spatiotemporal gait parameters accounted for by variance in the independent variables; *β* = standardized regression coefficient; *p value* = Significance of *β*. * *p* values < 0.05.

## Discussion

In the present study, we investigated the association between the toe flexor strength and spatiotemporal gait parameters. After adjustment, the associations were found only in the fast-pace walking condition. Decreased strength of toe flexor was correlated with slower walking speed, shorter periods of single-limb support phase (represented by lower percentage of swing time in gait cycle), and shorter stride length during fast-pace walking. Slower walking speed is a sensitive marker of mobility limitation in older adults, and shorter periods of single-limb support phase represent poor balance control during gait [23, 24]. Thus, these change of spatiotemporal gait parameters indicate deterioration in walking ability. Our results suggest that toe flexor muscles play an important role in walking at a fast pace in older adults.

Fast-pace walking requires generation of high peak power and greater functional reserve compared with usual-pace walking [13, 25]. It was reported that total generated mechanical work of the lower extremities is more strongly affected by aging at the fast-pace walking condition compared with the usual-pace condition [15]. The toe flexor muscles exert the propulsive force when walking. Thus, walking at a usual pace may not sufficiently require the function of the toe flexor muscles, and the associations between the strength of toe flexor muscles and spatiotemporal gait parameters would be observed in only the fast-pace condition.

The findings of our study are similar to those by Spink et al. in terms of the finding that toe flexor strength was correlated with walking speed [10]. However, the authors reported that hallux plantar flexion strength was a significant and independent predictor of walking speed at normal pace. In the present study, toe flexor strength was associated with walking speed at a fast pace, but not at a usual pace. This discrepancy may depend on the sample characteristics, as Spink’s subjects may be frailer as shown by a slower than usual pace walking speed (our study sample: 1.42 ± 0.19 m/s *vs.* Spink sample: 0.96 ± 0.20 m/s) and by 11.48% of subjects using a walking aid outside of the home (in our study no subjects used a walking aid outside of the home). Therefore, walking at usual pace might be challenging for Spink’s subjects who had a lower physiological reserve required for function of the toe flexor muscles. Some of the settings in our study were also different from other reports. For example, we investigated the total strength of the toe flexor (hallux and lesser toes) using a foot-gripping force meter with a strain-gauge, while the study by Spink et al. investigated the strength of the hallux flexor and lesser toe flexor separately using hand-held dynamometry. We also performed an adjustment using the anthropometric index (body weight and body height) and other characteristic measures, which may result in our finding of toe flexor strength associated with walking speed at fast pace only.

Our results have potentially important implications for older people. To live an independent life, there are many situations requiring people to walk at faster speeds than usual-pace (e.g., while crossing a street to avoid a collision with an oncoming car or while making a hurried visit to a destination in time). Our results suggest that the maximum speed of community-dwelling older people with poorer toe flexor strength needed in such situations was smaller than that of older people with better strength. Furthermore, to our knowledge, this is the first study to indicate the associations between toe flexor strength and spatiotemporal gait parameters including periods of single-limb support phase and stride length, specifically in healthy community-dwelling older adults. We found that a decreased strength of toe flexor was associated with a shorter percentage of single-limb support phase and a shorter stride length during fast-pace walking. Periods of single-limb support phase are related to balance control [24]. Stride length is the major source to change walking speed [24]. These two parameters are fundamental measures that represent human walking features. Thereafter, older people with poor toe flexor strength may experience difficulty walking safely under certain conditions. These results provide further support for an important role of the toe flexor muscle.

There are several explanations for the association between toe flexor strength and spatiotemporal gait parameters. Toe muscle strength has been shown to be associated with balance function (e.g., maximum balance range or coordinated stability) [9, 10, 26]. Subjects with poor toe flexor strength may have more difficulties in maintaining their body stability, especially for single-limb support, which may result in the association between reduced toe flexor strength and shorter periods of single-limb support or slower walking speed. A higher strength of the toe flexors is required for higher push-off forces needed to propel the body farther forward. Menz et al. [12] reported that the plantar flexion strength of the hallux was associated with maximum force and peak pressure under the hallux during the propulsive phase of gait. Thus, a lower strength of toe flexor would be associated with lower push-off forces and lower propulsion of the body further forward, which is related to shorter stride length and slower walking speed.

Our study has some limitations. First, our findings are based on cross-sectional associations, and therefore they do not allow any inference of causal relationships. Future studies are warranted to establish the temporal direction of the relationships between the toe flexor strength and gait parameters. Second, although we were able to control for characteristics likely to modify this relationship, residual potential confounders still may be present, including cognitive function [20] or psychosocial function [27]. However, there are few studies that have investigated the association between gait parameters and strength of toe flexor considering the potential confounding factors. Thus, we believe our results indicating such associations after adjustments for confounding are important. Third, we did not conduct a randomized order of the two walk tests to eliminate a learning effect in completion of the second course (i.e., the fast-pace condition). Many other studies examining gait parameters during various walking speed conditions have used a validated protocol involving usual-pace walking performed first, following by other conditions including fast-pace walking [13, 20–22]. Therefore, we used this protocol in the present study.

## Conclusion

Decreased strength of the toe flexor was correlated with slower walking speed, shorter periods of single-limb support phase, and shorter stride length during fast-pace walking. These correlations provide further support for an important role of toe flexor muscles in walking. Additional research is required to establish whether intervention programs that include strengthening exercises for the toe flexor muscle may achieve improvement of spatiotemporal gait parameters.

## Authors’ original submitted files for images

Below are the links to the authors’ original submitted files for images. Authors’ original file for figure 1 Authors’ original file for figure 2

## Abbreviations

LSA: Life-space assessment
%swing time: Percent of swing time in gait cycle.
