# Supplementary figures and images for: Association between toe flexor strength and spatiotemporal gait parameters in community-dwelling older people
Source: J Neuroeng Rehabil. 2014 Oct 8;11:143. doi: 10.1186/1743-0003-11-143 (PMC4200236; doi:10.1186/1743-0003-11-143)

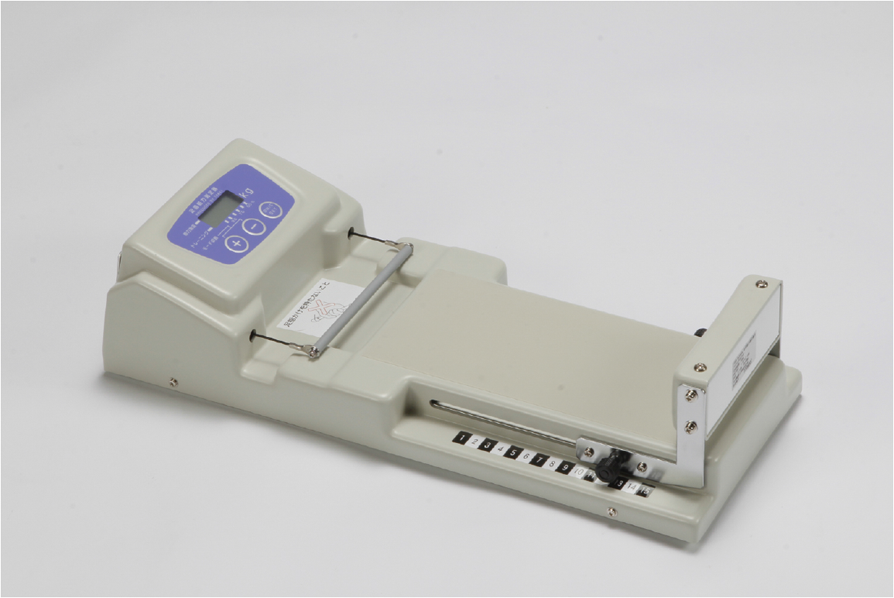

Supplement: Supplementary file 1 — Authors’ original file for figure 1 [file 12984_2014_664_MOESM1_ESM.tiff]

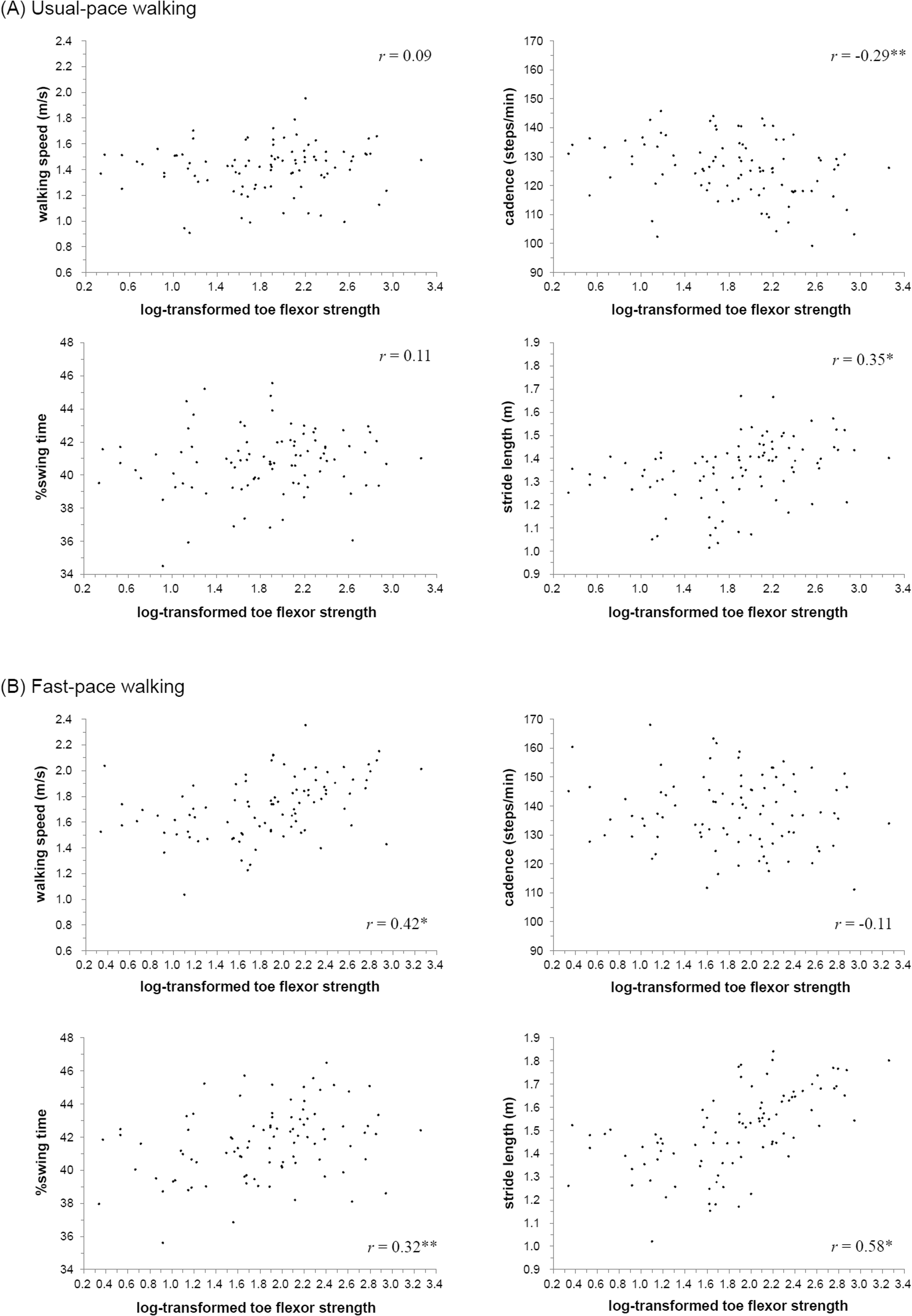

Supplement: Supplementary file 2 — Authors’ original file for figure 2 [file 12984_2014_664_MOESM2_ESM.tiff]
